# Artificial Intelligence for the Prediction of Preeclampsia: Current Evidence, Comparison with Conventional Screening Models, and Future Perspectives

**DOI:** 10.3390/diagnostics16182963

**Published:** 2026-09-13

**Authors:** Maria Fanaki, Dimitrios Baroutis, Panagiotis Antsaklis, Georgios Daskalakis, Vasileios Pergialiotis

**Affiliations:** First Department of Obstetrics and Gynecology, Division of Gynecologic Oncology, “Alexandra” General Hospital, National and Kapodistrian University of Athens, 11528 Athens, Greece; dbaroutis@gmail.com (D.B.); panosant@gmail.com (P.A.); pergialiotis@yahoo.com (V.P.)

**Keywords:** preeclampsia, artificial intelligence, machine learning, deep learning, biomarkers, angiogenic factors, ultrasound, Doppler, metabolomics, proteomics, precision obstetrics

## Abstract

Preeclampsia remains one of the leading causes of maternal and perinatal morbidity and mortality worldwide. Although current first-trimester screening strategies have improved risk assessment, their predictive performance remains limited by the biological complexity and heterogeneity of the disease. Artificial intelligence (AI) has emerged as a promising approach capable of integrating multidimensional clinical and biological data to improve early prediction. This review aims to summarize current evidence regarding AI-based prediction models for preeclampsia, compare their performance with conventional screening strategies, and discuss future directions for clinical implementation. A narrative review of published studies evaluating machine learning and deep learning models for first-trimester prediction of preeclampsia was performed. Studies incorporating maternal characteristics, hemodynamic variables, biochemical biomarkers, imaging, radiomics, and multi-omics data were reviewed. Diagnostic performance, predictor variables, and validation strategies were critically compared. Several studies have reported improved predictive performance of AI models compared with conventional statistical approaches, particularly when multimodal datasets were incorporated. High-performing models achieved area under the receiver operating characteristic curve (AUC) values ranging from 0.84 to 0.92. Across studies, maternal clinical characteristics, mean arterial pressure, uterine artery pulsatility index, placental growth factor, and pregnancy-associated plasma protein-A were the most consistently identified predictors. However, direct comparisons remain limited by methodological heterogeneity. Emerging approaches incorporating inflammatory biomarkers, cell-free nucleic acids, radiomics, and multi-omics technologies showed encouraging results but currently lack sufficient prospective multicenter validation for routine clinical implementation. AI has considerable potential to improve first-trimester prediction of preeclampsia, although prospective multicenter validation, standardized reporting, and implementation studies remain necessary before routine clinical adoption. Future research should prioritize prospective multicenter validation, standardized data collection, explainable AI, and seamless integration into clinical workflows to facilitate implementation in precision obstetric care.

## 1. Introduction

Preeclampsia is a multisystem hypertensive disorder unique to pregnancy and remains one of the most important causes of maternal and perinatal morbidity and mortality worldwide. Affecting approximately 2–8% of pregnancies, it contributes substantially to maternal deaths, preterm birth, fetal growth restriction, placental abruption, and stillbirth, while also imposing a considerable socioeconomic burden on healthcare systems, particularly in low- and middle-income countries where access to specialized maternal care may be limited [1]. Beyond its immediate obstetric consequences, preeclampsia is increasingly recognized as a disease with lifelong implications, predisposing affected women to chronic hypertension, cardiovascular disease, chronic kidney disease, and metabolic disorders, while offspring are at increased risk of cardiovascular and neurodevelopmental complications later in life [2].

Clinically, preeclampsia is characterized by the new onset of hypertension after 20 weeks of gestation accompanied by proteinuria or evidence of maternal organ dysfunction. However, the disorder represents a heterogeneous syndrome rather than a single disease entity, with substantial variation in its onset, severity, clinical manifestations, underlying pathophysiology, and pregnancy outcomes [3]. Early-onset preeclampsia, generally requiring delivery before 34 weeks of gestation, is primarily associated with defective placentation and severe placental dysfunction, whereas late-onset disease appears to be more closely related to maternal cardiovascular, metabolic, and inflammatory susceptibility factors [4]. This biological and clinical heterogeneity represents one of the greatest challenges to accurate risk prediction.

Early identification of women at increased risk for preeclampsia is essential, as preventive interventions such as low-dose aspirin initiated before 16 weeks of gestation can significantly reduce the incidence of preterm disease [5]. Consequently, several screening strategies integrating maternal characteristics, clinical history, biophysical measurements, and biochemical biomarkers have been developed. Although the risk assessment models proposed by NICE [6], ACOG [7], and the FMF [8] have significantly improved first-trimester screening, their predictive performance remains constrained by reliance on predefined variables and conventional statistical methods that may not adequately capture the complex, nonlinear interactions underlying disease development. Moreover, the increasing availability of electronic health records, advanced imaging, and high-throughput molecular technologies has generated multidimensional datasets that exceed the analytical capabilities of traditional prediction models.

In recent years, several systematic and narrative reviews have summarized the application of artificial intelligence in obstetrics and preeclampsia prediction [9]. However, most have focused primarily on the technical performance of machine learning algorithms or on selected biomarker categories, without comprehensively integrating conventional screening approaches, clinical characteristics, Doppler ultrasound, biochemical biomarkers, imaging, radiomics, and emerging multi-omics technologies into a unified clinical framework. Furthermore, few reviews have critically examined which predictors are consistently associated with high-performing AI models or discussed how these findings may be translated into routine first-trimester screening.

Therefore, the aim of the present narrative review is to provide an updated synthesis of the current evidence, compare AI-based prediction models with conventional screening strategies, identify the predictors most consistently associated with robust predictive performance, and propose an evidence-based multimodal framework for future first-trimester prediction of preeclampsia.

## 2. Methods

This narrative review was conducted to summarize current evidence regarding artificial intelligence for first-trimester prediction of preeclampsia. A literature search was performed using PubMed/MEDLINE, Scopus, and Web of Science. Searches included studies published up to May 2026 using combinations of the following MeSH terms: “preeclampsia”, “artificial intelligence”, “machine learning”, “deep learning”, “prediction”, “screening”, “placental growth factor”, “Doppler”, “radiomics”, and “multi-omics”.

Original studies evaluating AI-based prediction models, imaging approaches, biomarkers, radiomics, and multi-omics technologies relevant to preeclampsia prediction were considered. Priority was given to studies reporting diagnostic performance and external validation. Additional relevant references were identified from the reference lists of selected publications. As this article is a narrative review, a formal systematic review methodology or the PRISMA framework was not applied.

During the preparation of this manuscript, the authors used ChatGPT, GPT-5.6 Sol (OpenAI, San Francisco, CA, USA) for language editing, improvement of readability, and assistance with the presentation of selected graphical concepts. All AI-assisted content was critically reviewed, verified, and revised by the authors, who take full responsibility for the accuracy, integrity, and final content of the manuscript.

## 3. Conventional Prediction Models for Preeclampsia

**Figure 1:**
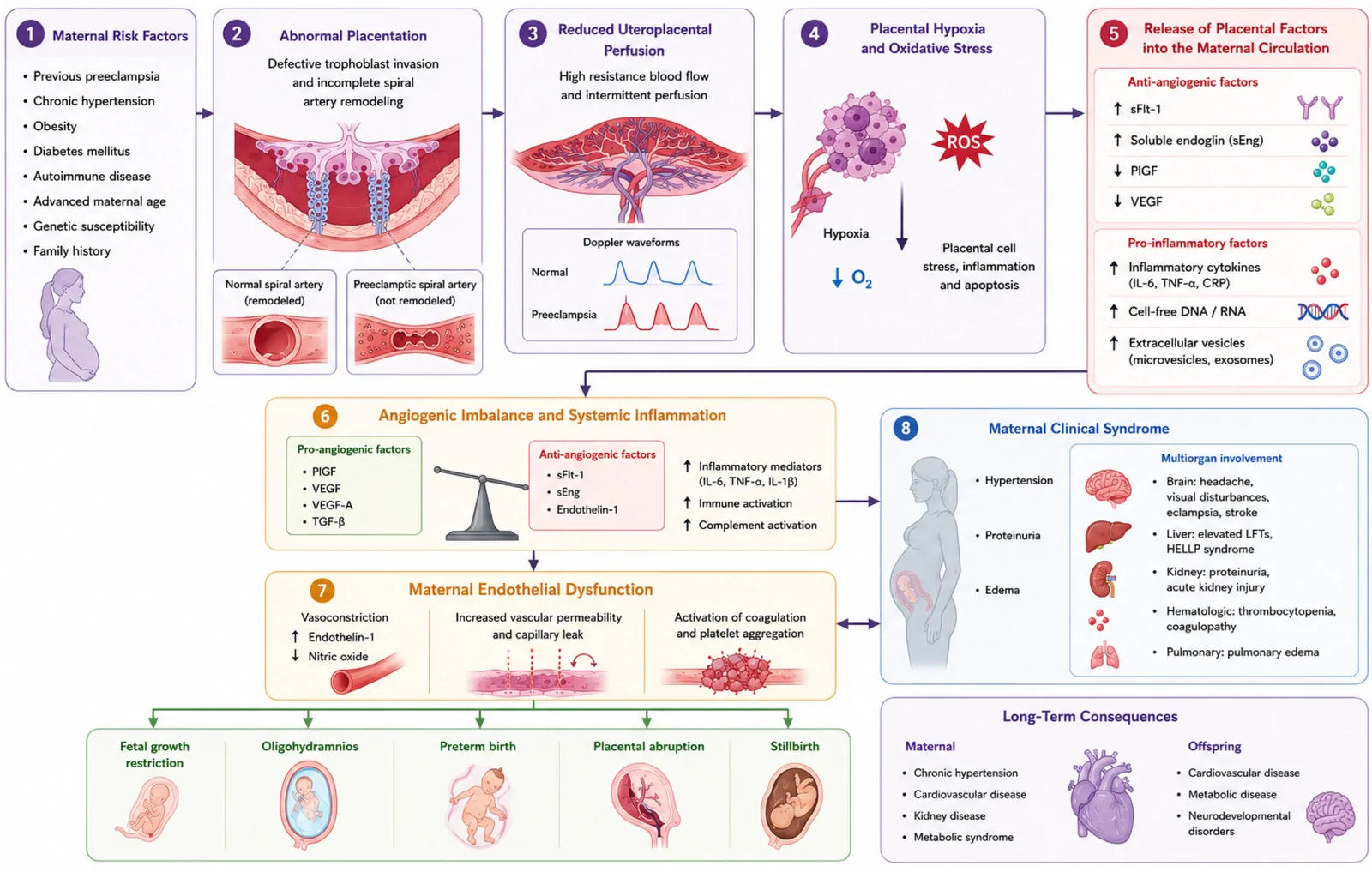
Pathophysiological mechanisms of preeclampsia.

Early identification of women at increased risk for preeclampsia is a cornerstone of modern obstetric care, as timely preventive interventions, particularly low-dose aspirin administration before 16 weeks of gestation, can significantly reduce the incidence of preterm disease [10]. Over the past decades, several conventional screening approaches have been developed based on maternal characteristics, clinical history, biophysical measurements, and biochemical biomarkers. Although these models have improved risk stratification, their predictive performance remains variable and is often influenced by population characteristics, disease prevalence, and resource availability. The rationale underlying contemporary prediction models is closely linked to the pathophysiological cascade of preeclampsia. Defective trophoblast invasion results in impaired spiral artery remodeling, placental hypoperfusion, angiogenic imbalance, oxidative stress, systemic inflammation, and widespread endothelial dysfunction. These mechanisms explain why current screening strategies incorporate maternal characteristics, Doppler indices, angiogenic biomarkers, and, increasingly, multi-omics data that reflect different stages of disease development (Figure 1). Together, these complementary data sources provide the biological foundation for both conventional prediction models and the more advanced AI-driven approaches discussed later in this review.

Historically, preeclampsia screening relied primarily on maternal demographic and clinical characteristics. Several maternal factors have consistently been associated with an increased risk of disease development, including a history of preeclampsia, chronic hypertension, pregestational diabetes mellitus, chronic kidney disease, autoimmune disorders, obesity, advanced maternal age, nulliparity, multiple gestation, and a family history of preeclampsia [11]. Risk assessment based solely on maternal characteristics is attractive because it is inexpensive, universally available, and easily implemented during the first prenatal visit. However, its predictive performance is relatively modest, particularly for early-onset preeclampsia. Most clinical risk-based approaches demonstrate low sensitivity and high false-positive rates, limiting their effectiveness as standalone screening tools.

To improve consistency and facilitate clinical decision-making, several professional societies subsequently developed standardized risk-based screening algorithms. The most widely adopted clinical screening strategies are those proposed by the National Institute for Health and Care Excellence and the American College of Obstetricians and Gynecologists. The NICE guidelines classify women as high-risk or moderate-risk based on maternal characteristics and recommend aspirin prophylaxis when at least one high-risk factor or two moderate-risk factors are present [12]. Similarly, ACOG recommends aspirin prophylaxis for women with established high-risk factors and selected combinations of moderate-risk characteristics [7]. These approaches are simple and require no specialized testing. However, because they rely exclusively on predefined clinical variables, their ability to identify women who will subsequently develop preeclampsia remains limited. Numerous studies have demonstrated that risk-factor-based screening fails to identify a substantial proportion of affected pregnancies, particularly among women without obvious clinical risk factors [13].

A major advance in preeclampsia prediction was achieved through the development of the screening algorithm proposed by the Fetal Medicine Foundation (FMF). Unlike traditional risk-based approaches, the FMF model integrates maternal characteristics with biophysical and biochemical markers obtained during the first trimester. The algorithm combines maternal demographic factors, mean arterial pressure (MAP), uterine artery pulsatility index (UtA-PI), placental growth factor (PlGF), and pregnancy-associated plasma protein-A (PAPP-A) within a competing-risks framework to estimate individualized risk. This multifactorial approach significantly improves screening performance compared with clinical risk assessment alone.

The FMF model currently represents the most extensively validated conventional screening strategy and forms the basis of first-trimester screening programs in many centers worldwide. Detection rates of approximately 75–90% for preterm preeclampsia have been reported when a 10% false-positive rate is accepted. Furthermore, the landmark ASPRE trial demonstrated that identifying high-risk women through FMF screening and administering low-dose aspirin significantly reduced the incidence of preterm preeclampsia [14]. Despite its superior performance, implementation of the FMF algorithm requires access to trained sonographers, biomarker testing, and specialized software, which may limit widespread adoption in resource-constrained settings. The incorporation of angiogenic biomarkers and Doppler ultrasound into the FMF algorithm also highlighted the growing importance of objective measures of placental function in preeclampsia prediction.

Accordingly, biochemical biomarkers have become increasingly important components of preeclampsia prediction. Among them, angiogenic markers have attracted particular attention due to their direct involvement in disease pathogenesis. Placental growth factor (PlGF) is a proangiogenic molecule produced by the placenta, whereas soluble fms-like tyrosine kinase-1 (sFlt-1) acts as an antiangiogenic factor by binding and neutralizing PlGF and vascular endothelial growth factor. Alterations in the sFlt-1/PlGF axis can be detected before the onset of clinical symptoms and correlate with disease severity [15].

Additional biomarkers investigated for prediction include pregnancy-associated plasma protein-A (PAPP-A) [16], placental protein 13 (PP13) [17], inhibin A [18], activin A [19], soluble endoglin [20], and various inflammatory mediators. Although many biomarkers demonstrate promising associations with disease development, their predictive value as standalone tests remains limited. Consequently, they are most effective when incorporated into combined screening models.

Complementing biochemical biomarkers, Doppler ultrasound evaluation of uterine artery blood flow provides an indirect assessment of placental vascular development. Increased uterine artery pulsatility index and persistent early diastolic notching reflect impaired trophoblast invasion and inadequate spiral artery remodeling, both characteristic features of preeclampsia [21]. Abnormal uterine artery Doppler findings are associated with an increased risk of preeclampsia, particularly early-onset disease and fetal growth restriction. As a result, uterine artery Doppler assessment has become an integral component of many screening algorithms, including the FMF model. Nevertheless, Doppler measurements are subject to operator variability and require specialized training and equipment. Furthermore, predictive performance is generally insufficient when Doppler assessment is used in isolation. Taken together, these limitations underscore that no single clinical, biochemical, or imaging marker is sufficient to accurately predict preeclampsia. Rather, integrating complementary sources of information has become the cornerstone of modern screening strategies and provides the conceptual foundation for the artificial intelligence-based prediction models discussed in the following section.

## 4. Limitations of Conventional Prediction Models

The principal conventional screening approaches currently used for preeclampsia prediction, together with their main components, predictive performance, strengths, and limitations, are summarized in Table 1. Despite substantial progress, conventional prediction models remain constrained by several limitations. Most approaches rely on predefined variables and conventional statistical methodologies that assume linear relationships between predictors and outcomes. Such models may fail to capture the complex biological interactions underlying preeclampsia development.

In addition, predictive performance often varies across populations due to differences in ethnicity, baseline risk, healthcare systems, and biomarker availability. Although the FMF algorithm currently represents the most effective conventional screening strategy, even this model does not identify all pregnancies that will subsequently develop preeclampsia.

These limitations have stimulated growing interest in artificial intelligence and machine learning approaches capable of integrating large multidimensional datasets, identifying nonlinear associations, and generating more personalized risk estimates. Such technologies may represent the next major step toward precision prediction of preeclampsia.

## 5. Artificial Intelligence and Machine Learning in Obstetrics

The increasing availability of electronic health records, medical imaging, and molecular data has accelerated the application of artificial intelligence (AI) in obstetrics. AI refers to computer systems capable of performing tasks that typically require human intelligence, while machine learning (ML), a subset of AI, enables algorithms to learn patterns from data and generate predictions without explicit programming. Unlike many conventional prediction models, machine learning algorithms are particularly well suited to analyzing large, heterogeneous, and high-dimensional datasets while automatically identifying complex interactions among variables. Although modern statistical methods can also accommodate nonlinear effects and predictor interactions, AI approaches may offer advantages when integrating numerous heterogeneous clinical, imaging, laboratory, and molecular variables within a single predictive framework [22].

AI applications in obstetrics have expanded rapidly and include the prediction of gestational diabetes, preterm birth, fetal growth restriction, adverse neonatal outcomes, and hypertensive disorders of pregnancy [23,24]. Several characteristics make preeclampsia particularly suitable for AI-based prediction. The disease is highly heterogeneous and results from complex interactions among maternal, placental, genetic, immunological, and environmental factors. Furthermore, prediction models may incorporate diverse sources of information, including maternal demographics, clinical history, blood pressure measurements, laboratory biomarkers, ultrasound findings, angiogenic factors, and multi-omics data. The ability of machine learning algorithms to integrate multidimensional datasets and identify complex nonlinear relationships may offer additional flexibility for individualized risk modeling. However, whether these capabilities translate into clinically meaningful improvements over established screening approaches remains to be confirmed in direct, externally validated comparisons.

### 5.1. Evolution of Artificial Intelligence Models for Preeclampsia Prediction

The application of artificial intelligence (AI) to preeclampsia prediction has evolved considerably over the past decade, paralleling advances in computational methods, data availability, and biomarker discovery. Early efforts focused on artificial neural networks (ANNs) trained on relatively small datasets containing maternal demographic characteristics, clinical history, and blood pressure measurements. Although these studies demonstrated the feasibility of AI-based prediction, their clinical applicability was limited by small sample sizes, restricted predictor availability, and insufficient external validation [25,26,27]. Nevertheless, these pioneering investigations established the conceptual framework that machine learning algorithms could identify complex nonlinear relationships among risk factors that might not be adequately captured by conventional regression-based models.

The widespread adoption of electronic health records and increasing computational power facilitated the development of more sophisticated machine learning approaches during the late 2010s and early 2020s. During this period, algorithms such as random forests, support vector machines (SVMs), gradient boosting machines, and extreme gradient boosting (XGBoost) were increasingly applied to large clinical datasets. These models incorporated a broader range of variables, including maternal characteristics, laboratory parameters, blood pressure measurements, and pregnancy-related complications. Machine learning algorithms can accommodate nonlinear interactions and high-dimensional relationships and, in several studies, have demonstrated encouraging predictive performance. However, these findings do not establish general superiority over contemporary statistical approaches, particularly given the limited number of direct externally validated comparisons [28].

A major milestone in the field was the development of large-scale first-trimester screening models integrating maternal characteristics with established biophysical and biochemical markers. Ansbacher-Feldman et al. developed a machine learning model using data from more than 60,000 pregnancies and demonstrated predictive performance comparable to the established FMF competing-risk algorithm [29]. Importantly, this study showed that machine learning approaches could effectively integrate maternal factors, mean arterial pressure, uterine artery Doppler indices, and placental biomarkers while identifying complex interactions among predictors that may not be fully captured by conventional statistical frameworks. This work provided a proof of concept that AI-based screening could potentially complement or enhance current first-trimester risk assessment strategies.

Subsequent studies have focused on validating and refining these approaches across diverse populations. In a multicenter Asian cohort comprising 10,935 pregnancies, Nguyen-Hoang et al. externally validated a first-trimester machine learning model incorporating maternal characteristics, mean arterial pressure, uterine artery pulsatility index, and placental growth factor (PlGF). Following recalibration, the model achieved AUC values of 0.84, 0.77, and 0.80 for preterm, term, and overall preeclampsia, respectively, demonstrating robust performance across different populations and healthcare settings [30].

Similarly, Li et al. evaluated multiple machine learning algorithms in a prospective cohort of 4644 singleton pregnancies screened at 11–13 + 6 weeks of gestation. The best-performing model, a Voting Classifier, achieved an AUC of 0.884 for preterm preeclampsia and demonstrated performance comparable to that of the FMF screening algorithm. These findings suggest that machine learning models may provide an alternative framework for integrating conventional screening markers while simultaneously exploiting complex interactions among predictors [31].

More recently, researchers have explored the use of routinely collected clinical information without specialized biomarker testing. Shyu et al. demonstrated that an XGBoost model based on standard clinical variables achieved an AUC of 0.921, highlighting the potential utility of AI-based screening in resource-limited settings where access to biochemical testing may be restricted [32]. Collectively, these developments illustrate the progressive transition from small proof-of-concept neural network models to large-scale, externally validated machine learning systems capable of integrating clinical, biochemical, and imaging data. This evolution has laid the foundation for the next generation of AI-based prediction models, which increasingly incorporate angiogenic biomarkers, multi-omics technologies, and advanced deep learning approaches to further improve early detection of preeclampsia.

Although these studies consistently demonstrate encouraging predictive performance, several methodological limitations should be considered. Many models were developed using retrospective datasets, outcome definitions varied considerably, and only a minority underwent external validation. Moreover, differences in predictor selection, gestational age at screening, and evaluation metrics limit direct comparison across studies. Consequently, reported performance should be interpreted cautiously until validated prospectively in diverse populations.

### 5.2. AI Models Based on Angiogenic and Biochemical Biomarkers

The incorporation of biochemical and angiogenic biomarkers into artificial intelligence (AI) frameworks represents one of the most important advances in contemporary preeclampsia prediction. While conventional screening models such as the Fetal Medicine Foundation (FMF) algorithm already integrate selected biomarkers, including placental growth factor (PlGF) and pregnancy-associated plasma protein-A (PAPP-A), machine learning approaches provide the opportunity to analyze multiple biomarkers simultaneously and identify complex nonlinear interactions that may not be captured by traditional statistical methods.

Among the most extensively investigated biomarkers are placental growth factor (PlGF), pregnancy-associated plasma protein-A (PAPP-A), soluble fms-like tyrosine kinase-1 (sFlt-1), and the sFlt-1/PlGF ratio [15]. These markers reflect the angiogenic imbalance that develops during abnormal placentation and can become abnormal weeks before the clinical manifestation of disease. Several machine learning studies have consistently identified PlGF as one of the strongest contributors to prediction performance. In a large population-based cohort of 4644 pregnancies, Li et al. demonstrated that PlGF and PAPP-A were among the most influential variables in machine learning models, with a Voting Classifier achieving an AUC of 0.884 for preterm preeclampsia prediction [31]. Notably, the relative contribution of these biomarkers differed between preterm and term disease, suggesting distinct biological pathways underlying different preeclampsia phenotypes.

Similarly, Ansbacher-Feldman et al. developed a neural-network model incorporating maternal characteristics, mean arterial pressure, uterine artery pulsatility index, PlGF, and PAPP-A. The addition of biomarkers increased the AUC from 0.816 to 0.909 and improved the detection rate for preterm preeclampsia from 53.3% to 75.3% at a 10% false-positive rate, highlighting the substantial predictive value of angiogenic markers within AI frameworks. Furthermore, feature-importance analyses identified PlGF as one of the most influential predictors, whereas PAPP-A contributed more modestly to model performance [29].

Additional evidence comes from Tabassum et al., who evaluated several machine learning algorithms using maternal characteristics together with first-trimester biomarkers. Their deep neural network achieved an accuracy of 93.4%, with PlGF, PAPP-A, and mean arterial pressure emerging as the most important predictive variables. These findings further support the biological relevance of angiogenic dysfunction in the early stages of disease development [33].

Recent studies have also expanded prediction models beyond traditional angiogenic biomarkers. Liang et al. developed a neural network model integrating inflammatory, hematological, and angiogenic markers, including PlGF, uterine artery pulsatility index, C-reactive protein (CRP), and the neutrophil-to-lymphocyte ratio (NLR). The model achieved an AUC of 0.917 in the development cohort and maintained good performance during external validation (AUC 0.838), suggesting that combining placental and inflammatory pathways may further improve predictive accuracy [34].

Beyond PlGF and PAPP-A, several additional biomarkers have shown promise. Soluble endoglin, activin A, inhibin A, interleukin-6, and interleukin-16 have all been associated with preeclampsia development and may provide complementary information regarding endothelial dysfunction and systemic inflammation [35,36,37]. Although their individual predictive value remains modest, machine learning algorithms offer the opportunity to integrate multiple biomarkers simultaneously and identify patterns that may not be apparent using conventional statistical methods.

Overall, current evidence suggests that biomarker-driven AI models may provide incremental predictive value beyond approaches based solely on maternal risk factors, particularly when angiogenic markers are incorporated. However, the magnitude and reproducibility of this benefit require confirmation in prospectively validated head-to-head studies. The most robust evidence currently supports the use of angiogenic markers, particularly PlGF and PAPP-A, while emerging data indicate that inflammatory and endothelial biomarkers may further enhance prediction when incorporated into multimarker machine learning frameworks.

Despite the promising performance of biomarker-based AI models, biomarker availability, laboratory standardization, assay variability, and differences in population characteristics remain important limitations that may affect reproducibility and generalizability. Furthermore, most inflammatory biomarkers have only been evaluated in relatively small cohorts and require prospective multicenter validation before incorporation into routine screening.

### 5.3. AI Models Incorporating Multi-Omics Data

Artificial intelligence has substantially expanded the application of multi-omics technologies for the prediction of preeclampsia. Unlike conventional biomarkers, which typically evaluate a limited number of predefined analytes, multi-omics approaches simultaneously interrogate thousands of molecular features across different biological layers, including genomics, transcriptomics, epigenomics, proteomics, metabolomics, lipidomics, and circulating cell-free nucleic acids. Machine learning algorithms are particularly well suited to these high-dimensional datasets because they can identify complex nonlinear interactions that are difficult to detect using traditional statistical techniques.

Among the most extensively investigated omics approaches is transcriptomic analysis of placental and maternal blood RNA. Placental dysfunction begins weeks before the onset of clinical symptoms, resulting in altered gene expression profiles that can be detected in maternal circulation [38]. Machine learning algorithms integrating uterine artery Doppler indices with maternal characteristics and biochemical markers have demonstrated promising discrimination and may capture complex interactions among predictors that are not readily represented in simpler models. Nevertheless, consistent superiority over established conventional screening approaches has not yet been demonstrated. Accordingly, several studies have shown that machine learning models incorporating cell-free RNA (cfRNA) signatures can accurately predict preeclampsia during the first trimester [39].

Closely related to cfRNA, cell-free DNA (cfDNA) provides another non-invasive molecular marker that reflects early placental dysfunction. Beyond its established role in prenatal aneuploidy screening, alterations in placental-derived cfDNA have been associated with trophoblast injury, placental ischemia, and abnormal placentation [40]. The fetal fraction, defined as the proportion of fetal-derived cfDNA relative to total circulating cfDNA, normally constitutes approximately 10–20% of total cfDNA during the first and second trimesters, whereas the remaining fraction is predominantly of maternal hematopoietic origin. Although both fetal-derived and total cfDNA concentrations increase in preeclampsia, the fetal fraction is paradoxically reduced, suggesting that the elevated circulating cfDNA primarily reflects increased maternal DNA release secondary to placental and systemic injury [41].

The integration of these cfDNA-derived features into AI models has shown encouraging results. In a large prospective multicenter study including 17,520 singleton pregnancies, Khalil et al. incorporated total cfDNA and fetal fraction into an artificial neural network for first-trimester prediction of preeclampsia. Women who subsequently developed preeclampsia had significantly higher total cfDNA concentrations (362.3 vs. 339.0 copies/mL) and a lower fetal fraction (7.5% vs. 9.4%; both *p* < 0.001). Although cfDNA variables had little impact on the prediction of overall preterm preeclampsia, their inclusion increased the sensitivity for early-onset preeclampsia by approximately 7%, indicating that cfDNA may provide complementary information when integrated with maternal clinical characteristics in AI-based prediction models [42]. Collectively, these findings suggest that circulating nucleic acid biomarkers, particularly when analyzed using machine learning approaches, offer valuable insight into placental health well before the onset of clinical disease.

Beyond circulating nucleic acids, metabolomics has also emerged as a promising approach for identifying early biochemical alterations associated with preeclampsia, reflecting disturbances in amino acid metabolism, lipid metabolism, mitochondrial function, and oxidative stress before the onset of clinical disease. Koster et al. identified stearoylcarnitine as a novel first-trimester biomarker that modestly improved conventional prediction models for early-onset preeclampsia, although the authors concluded that metabolomics-based screening was not yet suitable for routine clinical use [43]. Likewise, Prameswari et al. demonstrated significant alterations in maternal amino acid profiles in women with severe preeclampsia, including elevated phenylalanine, serine, glycine, and glutamate and reduced methionine, further supporting the role of metabolic dysregulation in disease pathogenesis [44]. Despite these encouraging findings, metabolomic biomarkers have not yet been widely incorporated into dedicated AI-based models for first-trimester prediction of preeclampsia.

Similarly, microRNAs (miRNAs) represent another promising class of molecular biomarkers because they regulate post-transcriptional gene expression and play essential roles in trophoblast proliferation, invasion, angiogenesis, immune regulation, and placental vascular remodeling. Aberrant expression of numerous circulating miRNAs, including placenta-specific members of the chromosome 19 microRNA cluster (C19MC), has been associated with impaired trophoblast invasion, oxidative stress, endothelial dysfunction, and the subsequent development of preeclampsia. In particular, dysregulation of miRNAs such as miR-210, miR-155, miR-515-5p, miR-486-5p, miR-455-3p, and members of the C19MC family has consistently been linked to abnormal placentation and disease severity, highlighting their potential as early biomarkers [45]. Although circulating miRNA signatures have demonstrated encouraging diagnostic and predictive performance, their integration into dedicated AI- or deep learning-based models for first-trimester prediction of preeclampsia remains limited.

Collectively, these findings demonstrate the considerable potential of multi-omics technologies to improve the early prediction of preeclampsia by capturing the complex molecular alterations that precede clinical disease. While AI models incorporating cfRNA and cfDNA have already shown promising predictive performance, the integration of other omics layers—including metabolomics and miRNA profiling—remains limited. Future AI models integrating multi-omics and clinical data may improve early prediction of preeclampsia, although prospective validation is essential before clinical implementation.

Although multi-omics approaches offer substantial biological insight, they remain expensive, technically demanding, and insufficiently standardized for routine clinical practice. Most published studies remain exploratory and lack prospective validation, limiting immediate clinical applicability.

### 5.4. Artificial Intelligence in Imaging and Ultrasound-Based Prediction of Preeclampsia

While molecular biomarkers provide insight into placental biology, ultrasound remains the cornerstone of first-trimester screening because it offers a non-invasive assessment of placental development and uteroplacental perfusion. AI has recently expanded the diagnostic potential of obstetric imaging by automatically extracting quantitative features from ultrasound examinations that may not be visually appreciable. These approaches combine conventional sonographic markers with advanced image analysis to improve the early prediction of preeclampsia.

Among ultrasound parameters, uterine artery Doppler has been the most extensively investigated. Elevated pulsatility index (UtA-PI), persistent early diastolic notching, and increased vascular resistance reflect impaired spiral artery remodeling and reduced uteroplacental perfusion, abnormalities that precede the clinical manifestation of preeclampsia [46]. Machine learning algorithms integrating uterine artery Doppler indices with maternal characteristics and biochemical markers consistently outperform conventional risk models by identifying complex nonlinear relationships between placental perfusion and disease development. A multicenter prospective validation study of 10,935 singleton pregnancies from seven Asian regions evaluated a machine learning model integrating maternal factors, MAP, UtA-PI, and PlGF for first-trimester prediction of preeclampsia. After retraining to account for assay-specific differences in PlGF measurements, the model achieved AUCs of 0.84 for preterm preeclampsia, 0.77 for term preeclampsia, and 0.80 for overall preeclampsia, demonstrating performance comparable with the established Fetal Medicine Foundation (FMF) competing-risk model for preterm and term disease. Importantly, women classified as high risk by the AI model but not by the FMF model had significantly higher UtA-PI values (median MoM 1.41 vs. 1.09 for preterm preeclampsia, 1.20 vs. 1.06 for term preeclampsia, and 1.31 vs. 1.04 for overall preeclampsia; all *p* < 0.001), indicating that machine learning algorithms may derive additional predictive value from uterine artery Doppler measurements beyond conventional statistical approaches [30].

Beyond conventional ultrasound biomarkers, radiomics has emerged as a novel AI-based imaging approach that extracts hundreds of quantitative features describing tissue texture, shape, intensity, and heterogeneity that are imperceptible to visual assessment. These imaging signatures provide objective measures of placental structural abnormalities associated with impaired placentation and vascular dysfunction. Unlike metabolomics and miRNA profiling, radiomics has already been incorporated into machine learning and deep learning models for preeclampsia. Liu et al. developed a radiomics nomogram based on diffusion-weighted brain MRI and apparent diffusion coefficient (ADC) maps that combined 11 radiomic features with maternal age and BMI, achieving AUCs of 0.93 in the training cohort and 0.89 in the validation cohort for distinguishing preeclampsia from gestational hypertension [47]. More recently, Zheng et al. proposed a multicenter deep learning radiomics (DLR) framework using automated placental segmentation on T2-weighted MRI in 420 pregnancies. The integrated DLR model outperformed conventional radiomics and deep learning alone, achieving AUCs of 0.84–0.89 for identifying preeclampsia across internal and external validation cohorts and an AUC of 0.92 for detecting preeclampsia complicated by fetal growth restriction [48]. Although these studies demonstrate the considerable potential of AI-driven radiomics, current models have primarily focused on diagnosing established disease rather than first-trimester prediction. Future research should evaluate whether radiomic features derived from early placental ultrasound or MRI can be integrated with clinical, Doppler, biochemical, and multi-omics data to improve early risk stratification for preeclampsia.

**Figure 2:**
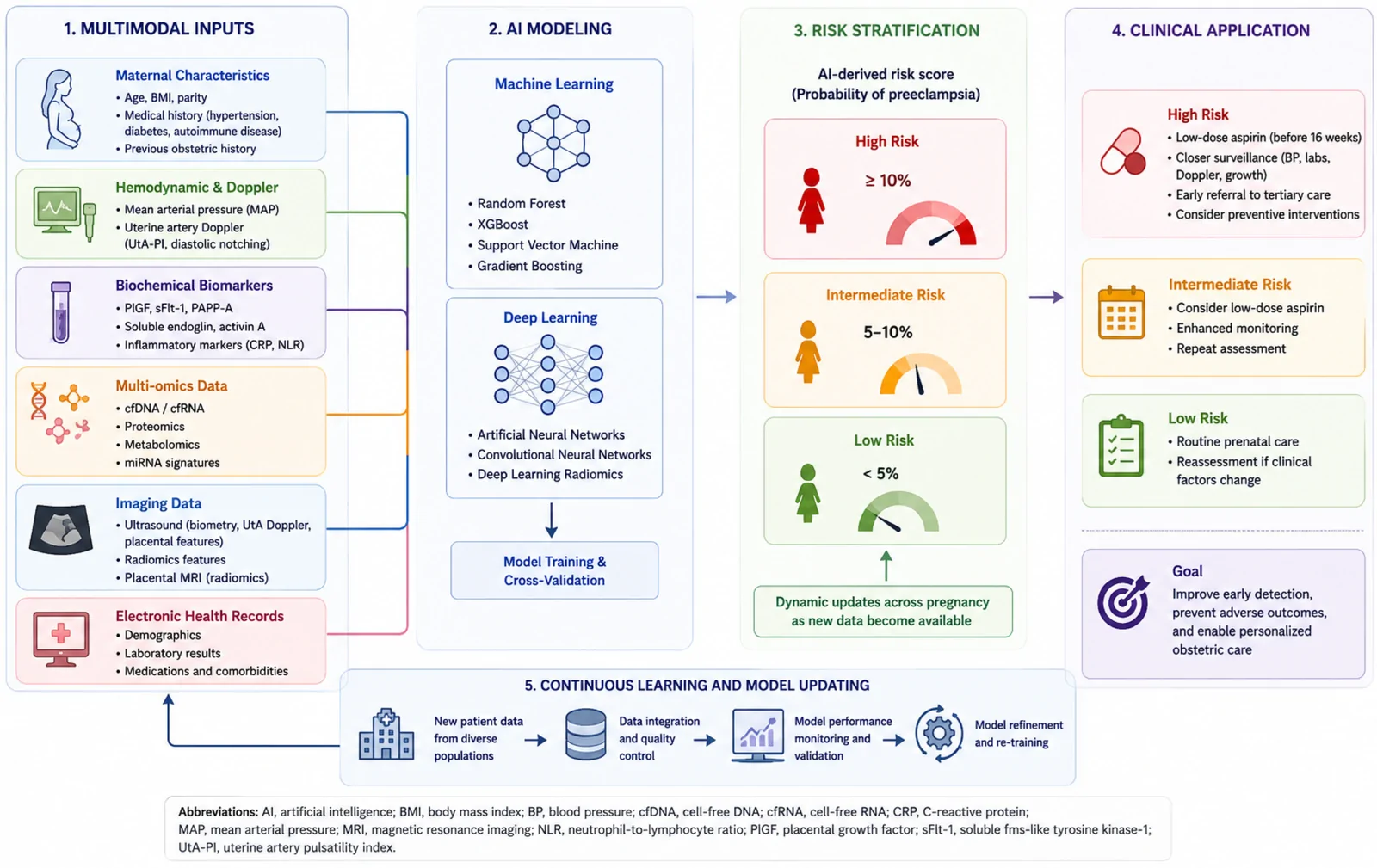
Integrated multimodal AI framework for early prediction of preeclampsia.

Overall, AI-enhanced imaging complements molecular biomarkers by providing quantitative assessment of placental structure and function. The integration of Doppler-derived hemodynamic parameters with advanced radiomic features represents a promising step toward more accurate and individualized prediction of preeclampsia. Nevertheless, larger prospective studies and external validation are required before these imaging-based AI models can be routinely implemented in clinical practice (Figure 2).

Collectively, current AI-based prediction models increasingly rely on the integration of heterogeneous clinical, biochemical, imaging, and molecular data to improve predictive performance. Although the algorithms differ in their computational approaches, predictor selection, and target outcomes, several have demonstrated high discrimination, particularly when multimodal datasets are incorporated. However, the extent of any incremental benefit over conventional prediction models remains uncertain because direct comparisons are limited and substantial methodological heterogeneity exists across studies. Representative studies, together with their algorithms, input variables, diagnostic performance, and validation status, are summarized in Table 2.

Importantly, radiomics-based prediction remains an emerging field. Most currently available studies evaluated established disease rather than true first-trimester prediction, and imaging protocols vary substantially between centers. Larger multicenter validation studies are therefore required before clinical implementation.

### 5.5. Proposed Evidence-Based Multimodal AI Framework for First-Trimester Prediction of Preeclampsia

The comparative analysis of representative AI-based prediction models (Table 2) demonstrates considerable consistency regarding the variables associated with the highest diagnostic performance, despite differences in study populations and machine learning architectures. Across the reviewed studies, predictive performance was determined primarily by the integration of complementary clinical and biological information rather than by the specific AI algorithm employed. Models incorporating multimodal datasets frequently reported high discrimination, with AUCs ranging from 0.84 to 0.92 across the reviewed studies.

However, these performance metrics should be interpreted cautiously. Reported AUC values cannot be directly compared across studies because they were obtained using different populations, outcome definitions, gestational ages at prediction, predictor sets, and validation strategies. In particular, externally validated models provide substantially stronger evidence of generalizability than models evaluated only using internal validation procedures.

Maternal clinical characteristics—including previous preeclampsia, chronic hypertension, maternal age, body mass index, diabetes mellitus, nulliparity, and multiple gestation—were incorporated into virtually all high-performing models and therefore represent the essential clinical foundation for risk assessment. Although these variables provide only moderate predictive performance when used alone, they consistently improved discrimination when combined with objective measures of placental function.

Among biophysical markers, mean arterial pressure (MAP) and uterine artery pulsatility index (UtA-PI) emerged as the most consistently utilized predictors. MAP was incorporated in nearly all first-trimester AI models achieving AUCs greater than 0.85, reflecting early maternal cardiovascular adaptation, whereas UtA-PI provides a quantitative assessment of impaired placental perfusion and spiral artery remodeling. Together, these parameters substantially enhanced predictive performance beyond maternal characteristics alone.

Among biochemical biomarkers, placental growth factor (PlGF) demonstrated the strongest and most consistent association with model performance. PlGF was included in almost every study reporting AUCs above 0.88 and represents the biomarker most directly reflecting early placental dysfunction. Pregnancy-associated plasma protein-A (PAPP-A) was the second most frequently incorporated biochemical marker and further improved discrimination when combined with MAP, UtA-PI, and PlGF. In contrast, sFlt-1, CRP, NLR, cfDNA, cfRNA, metabolomic signatures, and microRNAs showed encouraging results but were evaluated in fewer studies, exhibited greater methodological heterogeneity, and currently lack sufficient prospective multicenter validation for routine incorporation into first-trimester prediction models.

**Figure 3:**
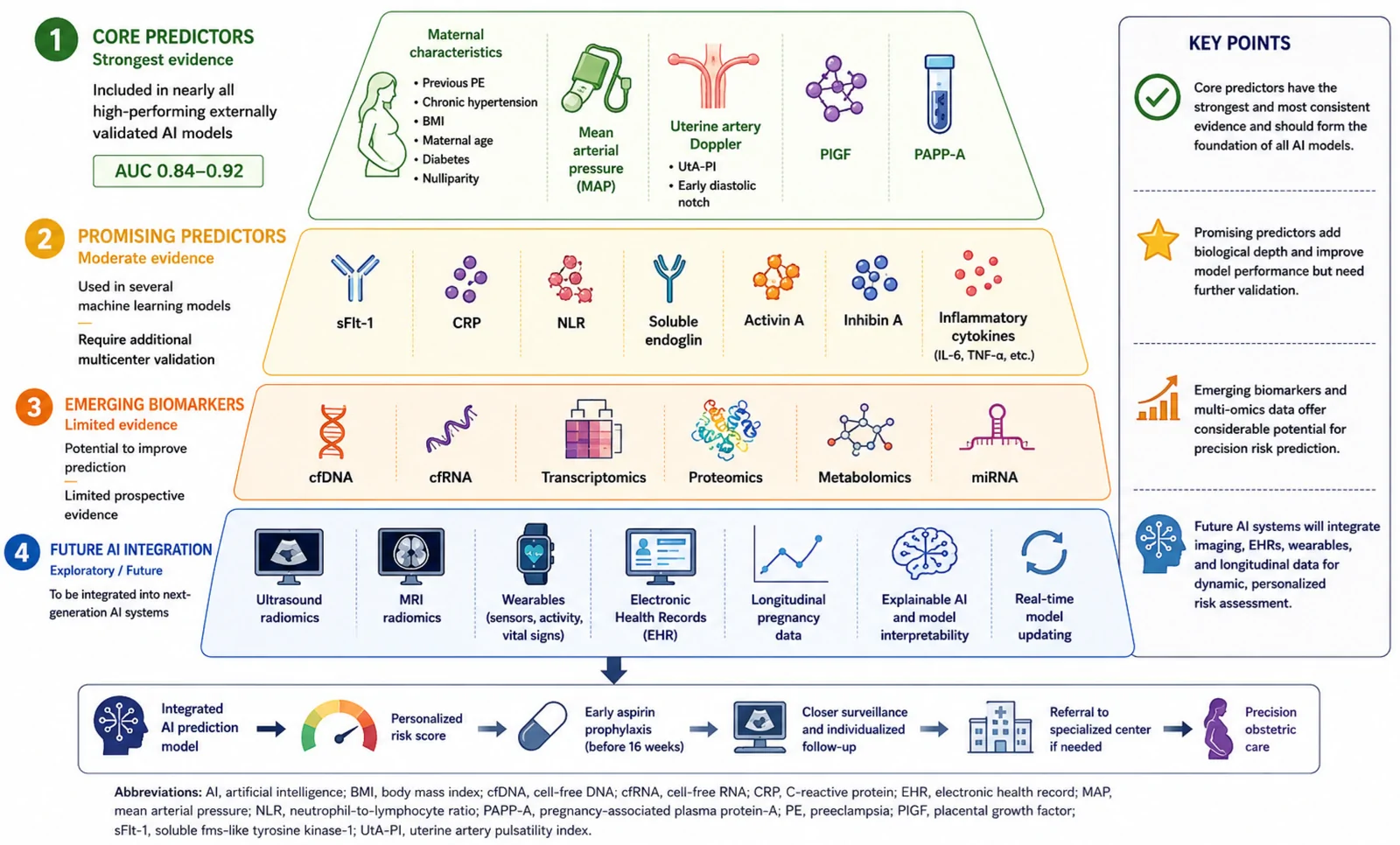
Evidence-based hierarchy of predictors used in artificial intelligence models for first-trimester prediction of preeclampsia.

Based on the available evidence, we propose a conceptual evidence-informed framework intended to summarize the predictor domains most consistently represented among high-performing AI models (Figure 3) comprising five core predictor domains: (i) maternal clinical characteristics, (ii) MAP, (iii) uterine artery Doppler (UtA-PI), (iv) PlGF, and (v) PAPP-A. This framework should not be interpreted as a validated clinical prediction model or as the definitive optimal predictor set but rather as a synthesis of the current literature that may guide future model development. These variables were selected because they were the most consistently represented among the highest-performing and externally validated models, collectively yielding AUC values ranging from 0.84–0.92. Additional inflammatory and multi-omics biomarkers may be incorporated as second-tier predictors in specialized settings as further evidence becomes available. Future AI models may also benefit from incorporating genomic information, including single-nucleotide polymorphism (SNP)-based susceptibility markers, together with clinical, biochemical, and imaging variables. Recent studies combining deep learning with genetic risk assessment suggest that genomic information may provide complementary predictive value and represents an important direction for future research [49].

Importantly, no single machine learning algorithm consistently outperformed the others. Artificial neural networks, deep neural networks, voting classifiers, XGBoost, random forests, and other ensemble methods all demonstrated excellent discrimination when trained using these core variables. Collectively, the available evidence suggests that appropriate predictor selection and high-quality multimodal data contribute more to predictive performance than the specific AI architecture, emphasizing the importance of standardized predictor sets and prospective external validation in future model development.

## 6. Clinical Implications

The rapid evolution of AI-based prediction models has important implications for contemporary obstetric practice. By integrating maternal characteristics, Doppler findings, biochemical biomarkers, imaging, and emerging multi-omics data, AI offers a more comprehensive assessment of preeclampsia risk than conventional screening strategies. Such models could support personalized antenatal care by identifying women who are most likely to benefit from early low-dose aspirin prophylaxis, intensified maternal and fetal surveillance, referral to specialized centers, and individualized follow-up. Importantly, AI should not be regarded as a replacement for established clinical assessment but rather as a decision-support tool capable of enhancing existing screening strategies through potentially more refined and individualized risk stratification. As electronic health records and automated ultrasound analysis become increasingly integrated into routine clinical practice, AI-driven prediction models may facilitate real-time risk assessment and more efficient allocation of healthcare resources.

Importantly, first-trimester prediction models should not be viewed exclusively as tools for identifying women at risk of preeclampsia. Rather, they represent a broader strategy for detecting impaired placental development and placental dysfunction early in pregnancy. Abnormal placentation underlies several major obstetric complications, many of which overlap biologically and clinically with preeclampsia. Consequently, the same maternal, biophysical, biochemical, and imaging markers incorporated into contemporary AI-based prediction models may also identify pregnancies at increased risk of fetal compromise, spontaneous preterm birth, medically indicated (iatrogenic) preterm or term delivery due to maternal or fetal indications, and fetal growth restriction (FGR) or small-for-gestational-age (SGA) neonates. Future AI models integrating multimodal placental biomarkers may therefore facilitate comprehensive risk stratification for placenta-mediated pregnancy complications rather than focusing exclusively on preeclampsia, thereby supporting more individualized antenatal surveillance and preventive strategies.

Despite these promising developments, several important knowledge gaps remain. Most AI models have been developed using retrospective datasets and have undergone limited prospective multicenter validation. Direct comparisons between different machine learning algorithms remain difficult because of heterogeneity in study populations, predictor selection, outcome definitions, gestational age at screening, and performance metrics. Furthermore, although emerging technologies such as multi-omics, radiomics, and wearable devices have shown considerable promise, their incremental clinical value beyond established biomarkers has not yet been fully established. Future research should therefore prioritize prospective validation, standardized reporting, harmonized data collection, and direct comparisons between AI-based and conventional screening strategies to determine the true clinical utility and cost-effectiveness of these approaches.

Beyond predictive performance, successful clinical implementation depends on several technical, ethical, and regulatory considerations. One of the principal limitations of many machine learning and deep learning algorithms is their limited interpretability. Unlike conventional regression models, where the contribution of individual predictors can be readily understood, complex AI algorithms frequently function as “black-box” systems, generating predictions without transparent explanations of how individual variables influenced the final risk estimate [50]. This lack of explainability may reduce clinician confidence and hinder adoption in routine obstetric practice, particularly when AI-derived predictions influence preventive interventions or decisions regarding maternal surveillance. Recent advances in explainable artificial intelligence (XAI), including SHapley Additive exPlanations (SHAP), Local Interpretable Model-Agnostic Explanations (LIME), attention mechanisms, and feature importance analyses, have sought to improve transparency by illustrating how individual variables contribute to model predictions [51]. These approaches may facilitate clinician acceptance and shared decision-making while maintaining high predictive performance.

Another major challenge concerns the generalizability and fairness of existing AI models. Although many algorithms demonstrate excellent discrimination during model development, relatively few have undergone rigorous external validation across geographically and ethnically diverse populations [52]. Differences in disease prevalence, healthcare systems, laboratory methodologies, ultrasound acquisition protocols, and population characteristics may substantially influence model performance when applied outside the original development cohort. Closely related to this issue is the risk of algorithmic bias, whereby AI systems trained on datasets that inadequately represent certain racial, ethnic, or socioeconomic groups may systematically underperform in these populations, potentially exacerbating existing disparities in maternal healthcare [53]. Future model development should therefore prioritize large multicenter collaborations, standardized data collection, and inclusion of diverse patient populations to improve robustness, equity, and generalizability.

Data quality represents another critical determinant of AI performance. Missing clinical information, inconsistencies in electronic health records, variability in laboratory assays, and operator-dependent ultrasound measurements may all reduce model reproducibility and predictive accuracy [54]. These challenges become even more pronounced when high-dimensional datasets such as transcriptomics, proteomics, metabolomics, radiomics, and other multi-omics technologies are incorporated into prediction models. Consequently, standardized acquisition protocols, rigorous quality-control procedures, and harmonized data-processing pipelines will be essential to ensure reproducibility and facilitate comparisons across studies.

The increasing use of large-scale clinical and molecular datasets also raises important ethical and regulatory considerations. AI-based prediction models require access to sensitive maternal health information derived from electronic health records, imaging repositories, and molecular databases, making robust data governance essential. Compliance with privacy regulations, including the General Data Protection Regulation (GDPR) and other national data protection frameworks, together with transparent policies regarding data ownership, informed consent, algorithm development, and accountability for AI-assisted clinical decisions, will be fundamental to maintaining public trust and ensuring responsible implementation [55]. Likewise, regulatory approval by agencies such as the United States Food and Drug Administration (FDA) and European medical device authorities will require evidence of analytical validity, clinical utility, safety, and ongoing performance monitoring, particularly for adaptive algorithms that continue to evolve after deployment.

Ultimately, the successful integration of AI into routine obstetric care will depend not only on algorithmic accuracy but also on seamless incorporation into existing clinical workflows. Integration with electronic health records, automated incorporation of laboratory and ultrasound findings, user-friendly interfaces, clinician education, interdisciplinary collaboration, and cost-effectiveness analyses will all be essential to facilitate widespread adoption. Rather than replacing clinical judgment, AI should function as an adjunctive decision-support tool that assists healthcare professionals in identifying women who may benefit from preventive interventions or intensified surveillance.

Collectively, these considerations indicate that the future of AI-based preeclampsia prediction extends beyond the development of increasingly accurate algorithms. Prospective multicenter validation, transparent and interpretable model design, standardized data collection, robust ethical governance, and effective clinical integration will all be essential to translate AI from promising research applications into reliable decision-support systems capable of improving maternal and neonatal outcomes.

## 7. Conclusions

Preeclampsia remains a major cause of maternal and perinatal morbidity and mortality, highlighting the need for accurate methods for early risk identification and timely preventive interventions. Although conventional screening strategies based on maternal characteristics, biophysical parameters, and biochemical biomarkers have substantially improved first-trimester risk assessment, their predictive performance remains limited by the complexity and heterogeneity of the disease.

Current evidence suggests that AI has considerable potential to improve individualized risk prediction, particularly through multimodal data integration. However, further prospective multicenter studies directly comparing AI-based and established screening strategies are required before definitive conclusions regarding superiority can be drawn. Machine learning and deep learning algorithms provide flexible approaches for modeling complex nonlinear relationships and integrating heterogeneous multimodal data, and several studies have reported encouraging predictive performance. Nevertheless, the available evidence is insufficient to conclude that these approaches are consistently superior to well-validated conventional screening models. Their principal potential may instead lie in their capacity to integrate diverse data sources and refine individualized risk estimation.

Machine learning and deep learning algorithms may facilitate the modeling of complex relationships and the integration of heterogeneous multimodal data; however, current evidence does not demonstrate consistent superiority over well-validated conventional screening approaches, and any incremental predictive benefit requires confirmation in direct, externally validated comparative studies.

Despite these encouraging findings, significant challenges remain before AI can be routinely implemented in clinical practice. Prospective multicenter validation, standardized data collection, improved model interpretability, mitigation of algorithmic bias, and robust regulatory oversight are essential to ensure reliable and equitable clinical application. Rather than replacing clinical judgment, AI should be viewed as a decision-support tool that complements existing screening strategies and assists clinicians in identifying women at increased risk for preeclampsia. Beyond improving the prediction of preeclampsia, future AI-based models may contribute to the early identification of the broader spectrum of placenta-mediated pregnancy complications, supporting precision obstetric care through comprehensive assessment of placental health.

As multimodal data integration, explainable AI, and computational methodologies continue to evolve, AI-based prediction models are expected to play an increasingly important role in precision obstetrics. Future efforts should focus on developing transparent, externally validated, and clinically applicable models capable of improving early prediction, guiding preventive interventions, and ultimately enhancing maternal and neonatal outcomes.

## Figures and Tables

**Table 1:** Comparison of conventional prediction methods for preeclampsia.

Screening Method	Main Variables	Timing of Assessment	Predictive Performance *	Advantages	Limitations
Maternal Risk Factors	Previous preeclampsia, chronic hypertension, diabetes, obesity, CKD, autoimmune disease, maternal age, parity, family history	First prenatal visit	Low–moderate (AUC ~0.60–0.70)	Simple, inexpensive, universally available	Low sensitivity and high false-positive rate; poor prediction of early-onset disease
NICE Guidelines	High- and moderate-risk maternal factors	First prenatal visit	Detection rate ~35–45% for preterm PE	Easy implementation; no additional testing required	Misses many women who subsequently develop PE
ACOG Guidelines	Maternal risk-factor assessment	First prenatal visit	Detection rate ~40–50% for preterm PE	Widely adopted; low cost	Limited predictive accuracy; relies solely on clinical factors
FMF Combined Screening Model	Maternal factors, MAP, UtA-PI, PlGF, PAPP-A	11–13 + 6 weeks	Detection rate 75–90% for preterm PE (10% FPR)	Best validated conventional model; individualized risk estimation	Requires biomarkers, Doppler ultrasound, and specialized software
Biochemical Biomarkers	PlGF, sFlt-1, PAPP-A, PP13, inhibin A, activin A, soluble endoglin	First–second trimester	Moderate (AUC ~0.65–0.85 depending on biomarker)	Objective and biologically relevant	Limited performance when used alone
Uterine Artery Doppler	UtA-PI, early diastolic notching	First–second trimester	Moderate (AUC ~0.65–0.80)	Non-invasive assessment of placental perfusion	Operator-dependent; insufficient as a standalone test

* Reported performance varies according to population characteristics, gestational age, outcome definition, and study methodology.

**Table 2:** Characteristics and diagnostic performance of representative AI-based prediction models for preeclampsia.

Author; Year	Study Population (*n*)	Algorithm	Input Variables	Prediction Target	Diagnostic Performance	External Validation
Ansbacher-Feldman et al.; 2022 [29]	>60,000	Artificial Neural Network (ANN)	Maternal characteristics, MAP, UtA-PI, PlGF, PAPP-A	Preterm preeclampsia	AUC: 0.909; Detection rate: 75.3% (10% FPR)	No
Nguyen-Hoang et al.; 2024 [30]	10,935	Machine learning model	Maternal characteristics, MAP, UtA-PI, PlGF	Preterm, term and overall preeclampsia	AUC: 0.84 (preterm); 0.77 (term); 0.80 (overall)	Yes
Li et al.; 2024 [31]	4644	Voting Classifier	Maternal characteristics, MAP, UtA-PI, PlGF, PAPP-A	Preterm preeclampsia	AUC: 0.884	No
Shyu et al.; 2025 [32]	NR	XGBoost	Routine clinical variables	Preeclampsia	AUC: 0.921	No
Tabassum et al.; 2025 [33]	NR	Deep Neural Network (DNN)	Maternal characteristics, MAP, PlGF, PAPP-A	Preeclampsia	Accuracy: 93.4%	No
Liang et al.; 2025 [34]	NR	Neural Network	PlGF, UtA-PI, CRP, NLR	Preeclampsia	AUC: 0.917 (development); 0.838 (validation)	Yes
Khalil et al. 2024 [42]	17,520	Artificial Neural Network (ANN)	Maternal characteristics, total cfDNA, fetal fraction	Early/preterm preeclampsia	Increased sensitivity for early preeclampsia (~7%)	Yes
Liu et al.; 2022 [47]	NR	Radiomics nomogram	MRI radiomic features, maternal age, BMI	Preeclampsia vs. gestational hypertension	AUC: 0.93 (training); 0.89 (validation)	Internal validation only
Zheng et al.; 2025 [48]	420	Deep Learning Radiomics	Placental MRI radiomic features	Preeclampsia; preeclampsia with fetal growth restriction	AUC: 0.84–0.89; AUC: 0.92 (PE + FGR)	Yes

## Data Availability

No new data were created or analyzed in this study. Data sharing is not applicable to this article.
